# L-T4 Therapy in Enteric Malabsorptive Disorders

**DOI:** 10.3389/fendo.2021.626371

**Published:** 2021-02-23

**Authors:** Poupak Fallahi, Silvia Martina Ferrari, Giusy Elia, Francesca Ragusa, Sabrina Rosaria Paparo, Alessandro Antonelli

**Affiliations:** ^1^ Department of Translational Research of New Technologies in Medicine and Surgery, University of Pisa, Pisa, Italy; ^2^ Department of Clinical and Experimental Medicine, University of Pisa, Pisa, Italy; ^3^ Department of Surgical, Medical and Molecular Pathology and Critical Care, University of Pisa, Pisa, Italy

**Keywords:** levothyroxine, hypothyroidism, enteric malabsorptive disorders, food interference, TSH

## Abstract

Levothyroxine (L-T4) absorption can be impaired by various causes: a) L-T4 ingestion during breakfast, or with food; b) conditions of reduced gastric acidity; c) intestinal procedures and diseases such as bariatric surgery, lactose intolerance (LI), celiac disease (CD), inflammatory bowel disease; d) drugs that alter L-T4 absorption, increasing the gastric pH, or preventing the dissolution of tablets. The development of new oral formulations, i.e. the liquid preparation and the soft gel capsule, represents the most recent advance regarding L-T4 therapy. Treating hypothyroidism with L-T4 tablets can lead to an improper control of thyroid-stimulating hormone (TSH) in ~10%–15% of patients. The improperly elevated TSH is usually managed by increasing the L-T4 daily dose, and revaluating TSH upon 2-6 months. The increase of the L-T4 dosage may cause iatrogenic hyperthyroidism, especially when the underlying disorders are cured. Liquid L-T4 can be administered in patients unable to swallow capsules or tablets, and this is one of its major benefits. Liquid L-T4 can: 1- overcome food and beverages interference; 2- bypass the malabsorption associated with an increased gastric pH; 3- circumvent the issue of malabsorption in patients who underwent bariatric surgery; 4-maintain TSH values under control better than L-T4 tablets in hypothyroid patients with typical or atypical CD, or in patients with LI. Few clinical studies evaluated soft gel L-T4 with encouraging findings in patients with gastric- or coffee-related malabsorption, or hypothyroid patients without malabsorption. Additional research is necessary to investigate liquid L-T4, or soft gel capsule, in other conditions of altered L-T4 absorption.

## Introduction

The synthetic hormone levothyroxine (L-T4) has a chemical structure comparable to T4, and it is prescribed as substitutive therapy of hypothyroidism-associated conditions (1). Its absorption occurs through the intestinal mucosa at the level of the duodenum, jejunum and ileum (2).

Firstly, L-T4 was isolated from porcine thyroid extracts (3), in 1914. Until the mid-1950s, desiccated thyroid extract was the only treatment of hypothyroidism; then synthetic L-T4 entered the market in the tablet formulation (4).

The frequency of hypothyroidism is higher in women, especially over 60 years, and its diagnosis can be done by measuring thyroid-stimulating hormone (TSH) and T4 levels by blood tests (1). Hypothyroidism is commonly caused by a low iodine intake in countries with severe iodine deficiency, and autoimmune thyroiditis, or treatment with radioiodine, or thyroidectomy, in developed countries (5–8). Drugs, such as tyrosine kinase inhibitors, are a novel cause of primary hypothyroidism (9).

At TSH-suppressive doses, L-T4 is used in patients with thyroid cancer to decrease/stop its growth (10), while in presence of nodular goiter its administration is controversial (11).

Since slight changes in blood levels can lead to treatment failure or iatrogenic thyrotoxicosis (12), the individualization of oral T4 treatment is necessary. The L-T4 daily dose is chosen according to the main cause of hypothyroidism, the therapeutical target [i.e. replacement or TSH suppressive treatment], and the patients’ body weight (13).

The supply of more sensitive TSH assays has progressively conducted to the reduction of L-T4 dose for replacement and TSH-suppressive treatments (14). The dosage of 1.5–1.7 µg/kg body weight/day is now considered as the optimal daily L-T4 replacement dose, which can normalize TSH levels in most hypothyroid patients (15). Despite this, about 20%–50% of patients do not respond to L-T4 (16) owing to different interfering issues (17), and need an increased dose, and monitoring (18). Some patients are not compliant with the prescribed regimen, causing a condition of pseudomalabsorption, and once excluded it, an altered intestinal absorption of L-T4, due to gastrointestinal disorders or interfering drugs, is considered the principal cause of refractory hypothyroidism (17).

In the era of precision medicine, therapies should be individualized and the characteristics of drugs should be evaluated accurately during a chronic treatment.

## L-T4 Tablet Malabsorption

A randomized, prospective study was performed to compare L-T4 ingestion after an overnight fast, 60 min prior to breakfast, or at breakfast. In case of L-T4 administered at breakfast, TSH was higher than while fasting, leading to the conclusion that it is advisable to ingest L-T4 in a fasting state, to avoid the interference on L-T4 tablet absorption caused by food and beverages (19).

Another study compared L-T4 ingestion with breakfast, versus 60 min before breakfast, versus bedtime. In this study it was shown that the 1 h before breakfast ingestion has not only lower TSH values but also substantially more uniform outcomes of TSH (20).

The lack of comparison of L-T4 ingestion 30 min, vs. 60 min before breakfast in the literature, suggests that this is an area of future research.

A study demonstrated that milk is also one of the interfering beverages that are frequently ingested with breakfast (21).

For example, L-T4 absorption is reduced if ingested 10 min before drinking coffee or with dietary fiber (22).

Since L-T4 tablets are composed by a stable salt, sodium L-T4, and different excipients, after its ingestion, the acid gastric pH is necessary to dissolve the tablet and remove sodium, to convert L-T4 into a lipophilic molecule (23, 24). The ingestion of L-T4 plus water improves drug absorption that is higher in the first 3 h, especially within the second hour (25). Approximately 70% of tablet L-T4 is absorbed (1).

Moreover, bariatric surgery can lead to medication malabsorption, in particular for L-T4 and cyclosporine, as shown in jejunoileal bypass, biliopancreatic diversion, and gastric bypass/gastroplasty (26).

Some drugs can alter L-T4 absorption, increasing the gastric pH [i.e. proton-pump inhibitors (PPIs), sucralfate, aluminum-containing antacids], preventing the dissolution of the tablet, others can bind L-T4 creating insoluble complexes (i.e. iron or calcium salts, ferrous sulfate, phosphate binders, calcium carbonate, bile acid sequestrants), while the mechanism of action of raloxifene is unknown (27).

Furthermore, various intestinal or gastric disorders, such as atrophic gastritis, Helicobacter pylori (HP) infection, celiac disease (CD), lactose intolerance (LI), and inflammatory bowel disease, can alter L-T4 tablet absorption (1).

## New Oral L-T4 Formulations

Patients refractoriness to a “normal dose” of L-T4 (27) has led to the development of novel hormonal preparations, the liquid formulation and soft gel capsule, to achieve an improved performance for this broadly advised drug.

### Liquid L-T4

The liquid preparation contains L-T4, ethanol, and glycerin, and it has the advantage that a gastric phase of dissolution of the tablet is not necessary (28), and it has a shorter mean time to attain the higher concentration than the tablets (29).

Also in newborns with congenital hypothyroidism, the reduction of TSH values observed with liquid L-T4 was higher vs. tablets (30, 31). Moreover, a first meta-analysis suggested that subjects receiving L-T4 tablets with suboptimal TSH values can achieve the desirable TSH after the switch to liquid L-T4 using the same dosage (32), and a second one indicated that the effectiveness of liquid L-T4 is higher (vs. tablets) in patients having or not having malabsorption in replacement or suppressive treatment (33).

Another study demonstrated a greater stability in the thyroid profile of hypothyroid elderly patients treated with liquid thyroxine as opposed to those being treated with tablet formulation over 5 years of follow-up (34). The state of pregnancy needs an adaptation of thyroid function, and for this reason hypothyroid women on replacement treatment with L-T4 usually require an increase of the dose of 30–50% at the beginning of pregnancy. A better control of serum TSH with the L-T4 liquid formulation was observed also in pregnant women (35). Thirty-one hypothyroid pregnant women who suffered from Hashimoto’s thyroiditis (HT) were involved in the study. Fourteen patients were in replacement therapy with liquid L-T4 and 17 were on tablets. Pre-pregnancy TSH and FT4 levels were similarly distributed in the two groups. During pregnancy, 8/31 (25.5%) of the women had to increase the dosage of L-T4. Among them, 7/17 (41.2%) were on L-T4 replacement therapy with tablets, and 1/14 (7.1%) with liquid L-T4. The mean dose of L-T4 from baseline to delivery was significantly increased only in women on tablets, as opposed to those on liquid therapy (35).

Furthermore, the effectiveness of liquid L-T4 was evaluated in hypothyroid patients with no malabsorption or drug interference. The stability of TSH levels was improved with liquid L-T4 with respect to tablets in these patients (36, 37).

The liquid L-T4 permitted to control better the stability of TSH levels also in patients who underwent thyroidectomy for cancer (38), or after bariatric surgery (39).

### Soft Gel Capsule

Sodium L-T4 is dissolved in water and glycerin in the soft gel capsule, and put in a gelatin matrix, to protect the active ingredient from degradation. It does not contain lactose, gluten, alcohol, sugar, or dyes (28).

A study evaluated whether the soft gel capsule formulation overcomes the malabsorption associated with the consumption of coffee, recruiting eight patients (including one with hypothyroidism, and the remaining seven patients under L-T4 suppressive doses in treatment for benign nodular goiter or recurrence of nodules after thyroidectomy) (40). The subjects were switched from tablets to capsule at the same L-T4 dosage for 6 months, with more consistent TSH outcomes (40).

A study investigated the daily requirement of L-T4 in 103 patients who underwent thyroidectomy, showing that the L-T4 requirement for attaining normal TSH values was not significantly different between soft gel capsules and tablets L-T4. However, although the TSH levels were within the normal range with both formulations, a statistically significant reduction of about 28% in the mean circulating TSH was observed with the soft gel formulation vs. tablets (41).

Another paper evaluated the new preparations of oral L-T4 in central hypothyroidism, reporting that liquid or soft gel L-T4, at the same dose as tablet L-T4, permit to obtain target serum FT4 levels above the midnormal range value (vs. tablets), suggesting the more favorable pharmacokinetics profile of either novel formulations in comparison with the tablet formulation (42).

Recently, a study evaluated the effect of switching from tablets to soft gel capsule in hypothyroid patients without an increased need of L-T4 (43). Circulating TSH level was in the normal range in 11/18 pts receiving L-T4 tablets, while after the switch in 16/18, and the median TSH was lower than that obtained with the classical formulation (43).

The above mentioned studies showed that liquid, and soft gel capsule L-T4 permit to maintain better the TSH stability (in the reference range) in hypothyroid patients.

## Liquid L-T4 and Food Interference

Liquid L-T4 can be administered in few patients who are unable to swallow capsules or tablets, and this is one of its major benefits. Thyroid hormones and TSH values were evaluated in subjects taking L-T4 in tablets, in comparison to patients receiving the liquid preparation with an enteral feeding tube, and it was shown that liquid L-T4 can be given by a feeding tube, facilitating its administration by nurses (31).

Other studies have investigated food interference with L-T4 absorption (44, 45). A placebo-controlled, double-blind, crossover, randomized trial enrolled 77 hypothyroid patients, who received randomly the liquid L-T4 at breakfast, or at least 30 min before it (46). TSH and thyroid hormones values were comparable in both cases, suggesting that the liquid L-T4 preparation can be swallowed at breakfast, in this way ameliorating the therapeutic compliance (46).

The lack of comparison of L-T4 ingestion 30 min, vs. 60 min before breakfast in the literature (even if guidelines suggest at least 30 min) encourages future research in this area.

## Liquid L-T4 Formulations in Disorders That Impair Gastric Acidity

Different gastrointestinal disorders, that interfere with the normal gastric acid secretory activity, decrease the efficacy of tablet L-T4 (47–49), and liquid L-T4 has been evaluated in these patients.

The acid environment of the stomach can be altered in presence of HP-associated gastritis, atrophic gastritis, or in patients who are taking PPIs (50).

The presence of HP influences drugs bioavailability, the level of gastric pH and of the inflammatory condition that occurs as a result of the disease itself (51, 52), leading to the uncertainty about the dosage that is absorbed. Patients with an altered acid secretion need an elevated dose of L-T4; the necessary daily dose was higher (of approximately 22–34%) in presence of atrophic gastritis, HP-related gastritis, or both (23).

A study compared the clinical effectiveness of tablet and oral liquid L-T4 in naïve hypothyroid patients with HP-infection. In patients with HP infection after 3 months (before HP eradication), subjects treated with liquid L-T4 showed a greater TSH reduction and a greater homogeneity in the TSH values, compared to L-T4 tablet. These results suggested that L-T4 liquid formulation may produce a better clinical response compared to the tablet formulation in hypothyroid subjects with HP infection (50).

L-T4 malabsorption is also a possible issue in presence of autoimmune atrophic gastritis (23, 53).

A study involved 391 patients with autoimmune thyroid disease administered with L-T4; 40% of them had positive parietal cell antibodies (PCA) and higher tablet L-T4 requirement in comparison to patients with negative PCA (1.24 ± 0.40 μg/kg vs. 1.06 ± 0.36 μg/kg). The required dose of tablet L-T4 was even higher in presence of an evident gastritis on histology (54).

A case series evaluated five patients with autoimmune gastritis, and hypothyroidism, receiving L-T4 tablets, who were switched to the same dosage of liquid L-T4. After the switch, circulating TSH normalized in all patients. In four of them, who were switched back to L-T4 tablets (same dose), TSH worsened, leading to the hypothesis that liquid L-T4 can circumvent the pH impairment associated with atrophic gastritis (55).

The secretion of gastric acid is blocked also by PPIs that bind covalently to the H^+^/K^+^ATPase enzyme (56). A crossover study, conducted in patients in whom tablet L-T4 absorption was impaired by PPIs, demonstrated a significant decrease in circulating TSH following the switch from the tablet preparation to the liquid one, at the same daily dose, maintaining the co-ingestion of PPI (57).

## Liquid L-T4 in Intestinal Malabsorption

### Bariatric Surgery

Drug malabsorption can derive from bariatric surgery (58).

Drug dissolution and solubility may be potentially altered in restrictive procedures that increase gastric pH in the newly created stomach.

In addition, highly lipophilic drugs are more likely to be affected because they are often dependent upon the availability of bile acids to enhance solubility. Often, these agents also undergo enterohepatic recirculation. Bypass of the upper small intestine limits the mixing of such drugs with bile acids to the common (post-anasatamotic) limbs of the distal small intestine. Jejunoileal bypass may also result in bile acid wasting (26).

Seventeen hypothyroid patients [who had been successfully treated with L-T4 tablets for more than 1 year prior surgery (13 Roux-en-Y gastric bypasses (RYGB); 4 biliary pancreatic diversions (BPD))] had elevated TSH levels from 3-8 months after surgery. Following the switch from tablets to liquid L-T4 (30 min prior to breakfast, same dose), TSH significantly decreased both in patients who underwent RYGB, or BPD, preventing the issue of malabsorption in BPD-treated patients, and in agreement with previous data obtained in patients who underwent RYGB. This leads to hypothesize that liquid L-T4 can bypass the malabsorption issue associated with bariatric surgery (39).

### Lactose Intolerance

LI should be evaluated among the gastrointestinal diseases leading to L-T4 malabsorption. In presence of LI, to restore euthyroidism or reduce the necessary dose of L-T4 (in tablets that usually contain lactose as an excipient), a low lactose diet, and/or a lactose-free L-T4 formulation, should be given (59, 60).

From 2009 to 2012, a cohort study was performed to analyze the replacement L-T4 dose (using tablets containing lactose) in 34 hypothyroid subjects with HT and LI, who were not compliant with a lactose-free diet (48). In presence of HT, the target TSH was achieved with a median L-T4 dose of 1.31 µg/kg/day. In patients with HT and LI, 5/34 reached the desired TSH with 1.29 µg/kg/day L-T4 (a similar dose). In the other 29 patients, the L-T4 dosage was gradually increased and the target TSH was achieved at a median L-T4 of 1.81 µg/kg/day (P<0.0001). In 6/29 patients, other gastrointestinal disorders were reported, and their median L-T4 dose was more elevated (2.04 µg/kg/day; P=0.0032). In the other 23/29 patients with LI, a median L-T4 dosage of 1.72 µg/kg/day (P<0.0001) was necessary to achieve target TSH levels. These data showed that in presence of LI the needed dose of L-T4 increased in a significant manner in hypothyroid patients (48).

The case of five patients with hypothyroidism and LI in treatment with an adequate dosage of L-T4 tablets (containing lactose) was reported, in whom the switch to a liquid L-T4 preparation (without lactose) at the same dosage caused the normalization of serum TSH. In 3 of them, TSH worsened again, after switching back to the tablets (61).

### Celiac Disease and Gluten Sensitivity

Another cause of **tablet** L-T4 malabsorption is CD, an immune-mediated enteropathy, developing in genetically susceptible subjects after the ingestion of wheat gluten, that can regress after the removal of gluten from the diet (62). Celiac patients with elevated TSH levels during L-T4 therapy showed an improvement after a gluten-free diet, thus revealing the importance of the alteration of the intestinal barrier in CD.

A study evaluated hypothyroid patients with concurrent CD. A total of 500 hypothyroid patients were enrolled and 29% of them needed a L-T4 dose ≥125 µg/day. CD was detected in nine patients. Eight/nine (89%) patients with CD required ≥125 µg/day of L-T4. Patients requiring ≥125 µg/day of L-T4 had a significantly higher risk of CD (P<0.001), and CD was found in 5.6% of them (63).

A study evaluated replacement tablet L-T4 dose in 35 patients with hypothyroidism, chronic autoimmune thyroiditis and atypical CD, as defined by the American Gastroenterological Association (47). In patients diagnosed with only HT, the target TSH level was attained after 5 ± 2 months of treatment with a median L-T4 dose of 1.31 μg/kg/day. Higher levels of TSH were observed in patients with HT and CD, following a comparable period and dose of L-T4. In 21 CD patients, target TSH was reached after 11 ± 3 months of gluten-free diet with no changes in the L-T4 dose. In the other 14 patients, noncompliant with the gluten-free diet, target TSH has also been attained but at higher L-T4 dosage (P=0.0002) than in hypothyroid patients without CD. These data suggested that atypical CD raises the need for L-T4, and the gluten-free diet or an increasing L-T4 dose can reverse this effect (47).

### Intestinal Parasitosis

Another cause of L-T4 malabsorption is chronic intestinal parasitosis. *Giardia lamblia* is one of the most prevalent human intestinal parasitic protist and can induce malabsorption of drugs and nutrients (causing anemia, weight loss, and growth retardation) (64). The case of a 57-year-old woman with hypothyroidism, well-controlled for the preceding 6 years, but unexpectedly showing poor hormonal control and abdominal symptoms, was reported. An adequate management of thyroidal function was obtained only after treatment of intestinal giardiasis (65). Moreover, the case of a 63-year-old woman with autoimmune hypothyroidism, well-replaced with tablet L-T4, who then became suddenly no more euthyroid, was shown (2). The patient reported the onset of acute gastrointestinal symptoms, associated with an increase in TSH levels. Owing to the suspect of a malabsorption disease, a switch from L-T4 tablets to a liquid formulation was suggested, to reach an optimal therapeutic target although the persistence of gastrointestinal symptoms, and thyroid hormones normalized. Further investigations permitted to diagnose a malabsorption syndrome due to *Giardia lamblia*, and liquid oral L-T4 solved the problem of its decreased absorption, even if the exact mechanism of action in the case of giardiasis is still not known (2).

These data suggest that giardiasis should also be considered in the assessment of the “difficult patient” with hypothyroidism, after ruling out non-adhesion and use of interfering drugs, in particular in areas, such as Brazil and other developing countries, with high prevalence of the infection, and in patients with symptoms of giardiasis (abdominal pain, diarrhea, weight loss, anemia) or with personal or family history of giardiasis (65).

## L-T4 Soft Gel Capsule in Gastroenteric Malabsorption

Few studies evaluated soft gel capsule preparation in enteric malabsorptive disorders. These preparations have been proven to have a different dissolution profile and absorption with respect to the traditional L-T4 tablet form by pharmacokinetic studies (24, 66).

Another study (67) compared L-T4 tablets and soft gel preparation in 31 patients with gastric-related L-T4 malabsorption. Inclusion criteria were: 1. patients with an increased need of thyroxine; 2. patients with a histologically diagnosed gastric disorder impairing gastric acid secretion. Patients took (since >2 years) L-T4 tablets, and then were switched to soft gel L-T4 capsule at a lower dosage (median reduction of T4 dose of 17%). Circulating TSH and FT4 were evaluated again in the following months. Even with the reduced dosage of L-T4, at each point of evaluation, median TSH levels were similar in 21 patients; whereas TSH was significantly higher in the remaining 10. It was concluded that a lower dosage of soft gel L-T4 capsule with respect to L-T4 tablet is necessary to preserve the therapeutic target in approximately 2/3 of subjects with altered gastric acid secretion (67).

The case of a woman with HT and hypothyroidism, in whom the altered absorption of L-T4 tablets, caused by PPIs, was corrected by the switch to soft gel capsule, was shown. Circulating TSH was lower than under the same dose of tablets. Switching back to tablets, serum TSH increased and then dropped when the dose was finally increased again (68).

A retrospective analysis was performed in 99 hypothyroid patients [of whom 24 reporting gastrointestinal comorbidity, in particular gastroesophageal reflux disease (11.1%), CD (6.1%), gastric bypass (3%)] who were switched from tablets to soft gel L-T4 capsule. Among the 99 patients studied, 51.5% had no documented changes in TSH status after the switch (P<0.0001), more than 26% of patients had a documented improvement in their TSH status (26/99), and only a minority of patients (22.2%) experienced a worsening of their TSH status post-switch. Improved hypothyroid symptom control was shown in 61.6% of patients (P<0.0001) (69).

## Conclusion

The development of new oral formulations (the liquid preparation, and the soft gel capsule) represents the most recent advance regarding L-T4 therapy. The treatment of hypothyroidism with L-T4 tablets can lead to an improper (elevated or suppressed) control of TSH in approximately 10-15% of patients (even with a correct L-T4 dosage) (1). The improperly elevated TSH, caused by the above reported conditions, is usually managed increasing the L-T4 daily dose, and a following re-evaluation of TSH upon 2–6 months. However, the increase of the L-T4 daily dosage may sometimes cause iatrogenic hyperthyroidism, in particular when the underlying disorders (i.e., a gluten free diet) are cured, or with the stop of interfering drugs.

Liquid L-T4 can be given to patients unable to swallow capsules or tablets, and this is one of its major benefits. Moreover, it has been reported that liquid L-T4: 1- overcomes the food and beverages interference, caused by food, coffee, or breakfast, observed with L-T4 tablets; 2- circumvents malabsorption induced by a raised gastric pH in atrophic gastritis, or due to PPI treatment, or in patients with HP infection; 3- can bypass the issue of malabsorption in patients who underwent bariatric surgery, or in patients with LI; 4- maintains better the TSH stability (in the reference range) in hypothyroid patients with a diagnosis of CD and gluten sensitivity, or intestinal parasitosis, even if properly treated for those conditions.

Soft gel L-T4 have been evaluated by few clinical studies, reporting encouraging data in patients with gastric- or coffee-related T4 malabsorption, or hypothyroid patients without malabsorption.

Further studies are ongoing to evaluate liquid L-T4, or soft gel L-T4, in other conditions of altered L-T4 absorption. Since enough data from randomized clinical trial are lacking, prospective, randomized studies are needed.

## Author Contributions 

PF, SMF, and AA conceived the paper. All authors contributed to the article and approved the submitted version.

## Conflict of Interest

The authors declare that the research was conducted in the absence of any commercial or financial relationships that could be construed as a potential conflict of interest.

The handling editor declared a past co-authorship with several of the authors: AA, FR, GE, PF, SMF, and SRP.
